# Orai1 Mediates Exacerbated Ca^2+^ Entry in Dystrophic Skeletal Muscle

**DOI:** 10.1371/journal.pone.0049862

**Published:** 2012-11-19

**Authors:** Xiaoli Zhao, Joseph G. Moloughney, Sai Zhang, Shinji Komazaki, Noah Weisleder

**Affiliations:** 1 Department of Physiology and Biophysics, Robert Wood Johnson Medical School, Piscataway, New Jersey, United States of America; 2 Division of Pharmacology, College of Pharmacy, Davis Heart and Lung Research Institute, The Ohio State University, Columbus, Ohio, United States of America; 3 Department of Anatomy, Saitama Medical University, Saitama, Japan; 4 Department of Physiology & Cell Biology, Davis Heart and Lung Research Institute, The Ohio State University, Columbus, Ohio, United States of America; University of Pittsburgh, United States of America

## Abstract

There is substantial evidence indicating that disruption of Ca^2+^ homeostasis and activation of cytosolic proteases play a key role in the pathogenesis and progression of Duchenne Muscular Dystrophy (DMD). However, the exact nature of the Ca^2+^ deregulation and the Ca^2+^ signaling pathways that are altered in dystrophic muscles have not yet been resolved. Here we examined the contribution of the store-operated Ca^2+^ entry (SOCE) for the pathogenesis of DMD. RT-PCR and Western blot found that the expression level of Orai1, the pore-forming unit of SOCE, was significantly elevated in the dystrophic muscles, while parallel increases in SOCE activity and SR Ca^2+^ storage were detected in adult *mdx* muscles using Fura-2 fluorescence measurements. High-efficient shRNA probes against Orai1 were delivered into the flexor digitorum brevis muscle in live mice and knockdown of Orai1 eliminated the differences in SOCE activity and SR Ca^2+^ storage between the *mdx* and wild type muscle fibers. SOCE activity was repressed by intraperitoneal injection of BTP-2, an Orai1 inhibitor, and cytosolic calpain1 activity in single muscle fibers was measured by a membrane-permeable calpain substrate. We found that BTP-2 injection for 2 weeks significantly reduced the cytosolic calpain1 activity in *mdx* muscle fibers. Additionally, ultrastructural changes were observed by EM as an increase in the number of triad junctions was identified in dystrophic muscles. Compensatory changes in protein levels of SERCA1, TRP and NCX3 appeared in the *mdx* muscles, suggesting that comprehensive adaptations occur following altered Ca^2+^ homeostasis in *mdx* muscles. Our data indicates that upregulation of the Orai1-mediated SOCE pathway and an overloaded SR Ca^2+^ store contributes to the disrupted Ca^2+^ homeostasis in *mdx* muscles and is linked to elevated proteolytic activity, suggesting that targeting Orai1 activity may be a promising therapeutic approach for the prevention and treatment of muscular dystrophy.

## Introduction

Muscular dystrophy is characterized by muscle degeneration and reduced contractile function due to the death of skeletal muscle fibers. The most common type, Duchenne muscular dystrophy (DMD), results from a loss of function of the dystrophin gene [1]. Dystrophin is a high molecular weight structural protein that stabilizes the sarcolemma of muscle fibers by linking cytoskeletal actin to laminin in the extracellular matrix through the dystroglycan complex [2], protecting the muscle against various mechanical stresses to maintain sarcolemmal integrity [3]. Additional studies indicate a role for dystrophin in modulating a number of different cellular processes and signaling events [4]. While the exact cause of muscle fiber death is not clearly established, there is an increasing body of evidence showing that a defect in Ca^2+^ homeostasis is a causal factor for the progressive damage observed in muscular dystrophy [5]. One of the early cellular defects observed in DMD muscle biopsies was an increase in the number of fibers positive for a histochemical Ca^2+^ staining [6], and later efforts established that DMD may be associated with increased influx of Ca^2+^ [7], [8]. However, the identity of the Ca^2+^ influx pathways that are altered in dystrophic muscle fibers has not yet been clearly resolved [9]–[14].

Store-operated Ca^2+^ entry (SOCE), or capacitative Ca^2+^ entry, was originally observed in non-excitable cells as a Ca^2+^ influx pathway stimulated by reduction of intracellular Ca^2+^ stores [15]. Previous studies from various investigators demonstrate that SOCE is present in skeletal muscle cells [16]–[18], and that SOCE plays an important function during stress conditions such as strenuous exercise and fatigue [19]–[22]. The molecular components of the SOCE machinery include stromal interaction molecule 1 (STIM1) as an endoplasmic/sarcoplasmic reticulum (ER/SR) Ca^2+^ sensor [23], [24] that translocates from the ER/SR membrane to regions close to the plasma membrane following depletion of the intracellular Ca^2+^ stores [25]. This movement of STIM1 activates Orai, a pore-forming unit that allows permeation of Ca^2+^ through the plasma membrane [26], [27] into the cytosol [28]. Recent studies indicate that Orai1 [29], [30] and STIM1 [31] comprise the principal isoforms composing the SOCE machinery in cultured myotubes and adult muscle fibers. In our previous study, we examined the mRNA expression levels of all known Orai isoforms and STIM1 and confirmed that Orai1 is the major isoform expressed in muscle cells while STIM1 is also abundantly expressed in muscle cells [32].

In this study, we compared the expression levels of SOCE components in wild type (*wt*) and *mdx* muscles using real-time PCR and western blotting. Our results showed that Orai1 was significantly upregulated in *mdx* muscle, while STIM1 levels remained largely unchanged. This observation was accompanied by a significant increase in SOCE activity and an elevated caffeine-sensitive SR Ca^2+^ store in the *mdx* muscle fibers. The contribution of Orai1 to aggravated SOCE in *mdx* fibers was confirmed by specific knockdown of Orai1 expression in adult skeletal muscle by a small hairpin (sh) RNA probe. Furthermore, treatment by BTP2, a specific SOCE inhibitor, significantly reduced the cytosolic calpain activity in dystrophic fibers. Our data establishes that Orai1 is an essential component of SOCE machinery in adult skeletal muscle and provides evidence to support that Orai1-mediated SOCE is a major pathway contributing to the elevated Ca^2+^ entry and increased proteolytic activity in dystrophic muscles.

## Results

**Figure 1:**
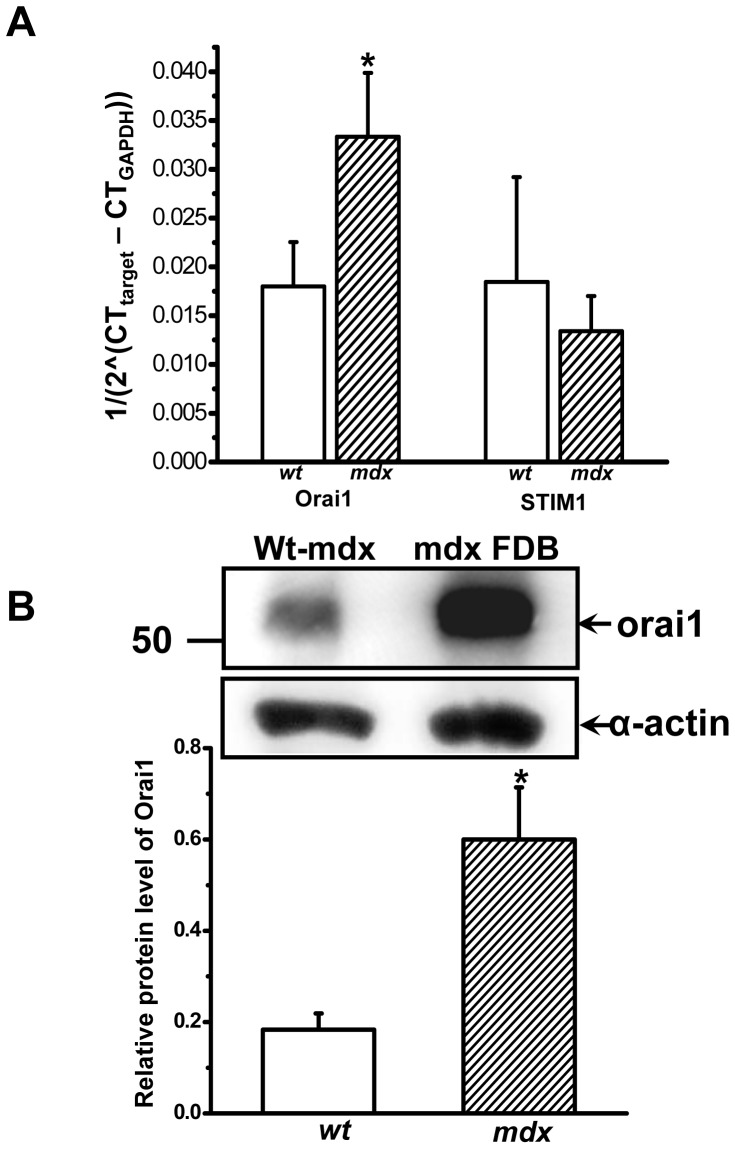
Up-regulation of Orai1 in *mdx* muscle. (**A**) mRNA expression levels of Orai1 and STIM1 in gastrocnemius muscles from *wt* C57BL/10ScSnJ and dystrophic C57BL/10ScSn-Dmd*^mdx^*/J mice were detected by real-time PCR. Relative mRNA copy numbers were shown, n = 6–8 for Orai1 and n = 4 for STIM1, ^*^ *P*<0.05. (**B**) Western blot showed upregulation of Orai1 protein (∼50 kD) in *wt* and *mdx* FDB muscles. The observed molecular size of Orai1 protein was higher than the predicted molecular weight of 33 kDa, possibly due to post-translational modification, splice variations or changes in relative charges of the amino acid of the protein. Sarcomeric α-actin (42 kD) was used as a loading control and for normalization of densitometry, n = 6∼7, ^*^ *P*<0.05.

Previous studies suggest a correlation between excessive Ca^2+^ entry and pathology in dystrophic muscle [13], [33]. However, the source of the aberrant Ca^2+^ entry in *mdx* muscle fibers remains unclear. SOCE has been shown to present in skeletal muscle cells [34], and previous studies identified the major components of SOCE as Orai1 [26] and STIM1 [24] in immune cells. Here we examined the expression levels of Orai1 and STIM1 in adult skeletal muscle from the *mdx* and strain- and age-matched *wt* mice (8 to 10 weeks old) using real-time PCR and western blot assays. Our initial screening of mRNA expression in gastrocnemius muscle shows the expression level of Orai1 was significantly higher in *mdx* muscle (**Fig. 1A**). Further studies show that the protein level of Orai1 is increased in flexor digitorum brevis (FDB) muscle (**Fig. 1B**) as well as gastrocnemius and extensor digitorum longus (EDL) muscle (**Fig. S1**), supporting the possibly that Orai1-mediated Ca^2+^ entry may be elevated in *mdx* muscle fibers.

**Figure 2:**
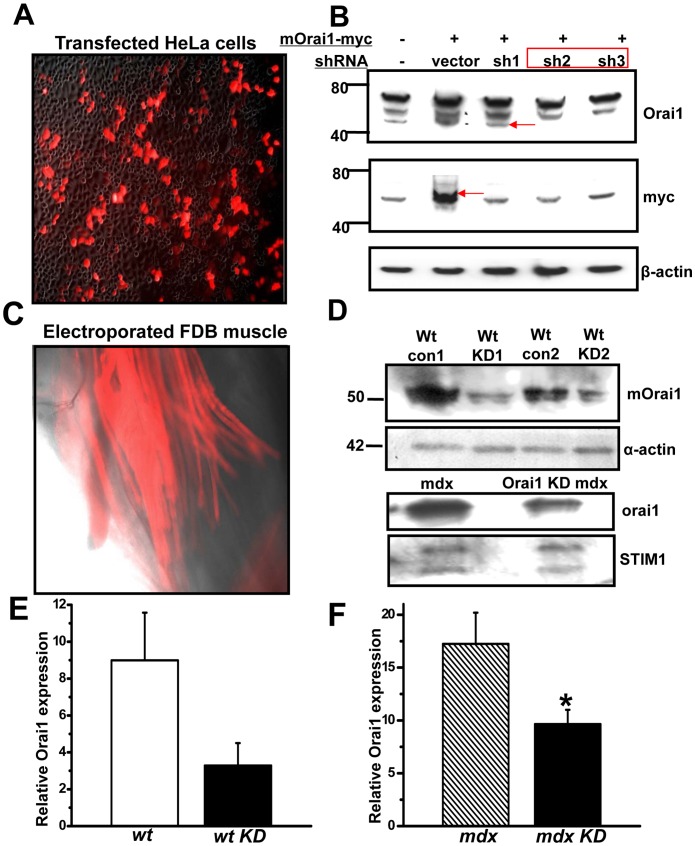
Effective knockdown of endogenous Orai1 gene in both *wt* and *mdx* FDB fibers by shRNA probe. (**A**) Superimposed transmission light and red fluorescent images (100x) of Hela cells after 24 h transfection with shOrai1 with a RFP marker and mouse full length Orai1 with a myc tag (mOrai1-myc). (**B**) Western blot showing effective knockdown of the exogenous mOrai1 gene by shOrai1 probes (sh1∼3) No. 2 and No. 3. β-actin was used as a loading control. (**C**) Fluorescence image (100x) showing the successful transfection of FDB muscle 2 weeks after electroporation of shOrai1 probe. (**D**) Single fiber western blots confirmed the effectiveness of shOrai1 in knocking down the endogenous mOrai1 in *wt* mice (upper panel) and *mdx* mice (lower panel). Pooled extracts from 15 individual FDB fibers were loaded per lane. The exposure used to generate these images was adjusted to produce a robust signal that could be used to assess the level of Orai1 knockdown for both *wt* and *mdx* mice. (**E**) Densitometry of Orai1 Western blot in wild type muscle transfected with control or shOrai1 vectors using NIH image J, n = 2. (**F**) Densitometry of Orai1 Western blot in mdx muscle transfected with control or shOrai1 vector, n = 3, *P<0.10.

To establish the direct link between Orai1 and store-operated Ca^2+^ entry in skeletal muscle, and to examine the status of Orai1-mediated SOCE in *mdx* muscle, we attempted to knock down Orai1 expression in native skeletal muscles. Three shRNA probes targeting the coding region of the full length mouse Orai1 cDNA (shOrai1) were designed and cloned into a vector containing a RFP marker cassette driven under a separate promoter. Efficacy of these shRNA probes were first tested in HeLa cells co-transfected with a myc-tagged full-length Orai1 cDNA (**Fig. 2A**). Our results revealed that all shRNA probes could effectively knock down the ectopic expression of mouse Orai1 with a myc tag (**Fig. 2B**). Two probes (sh2 and sh3) were chosen for subsequent functional studies. Blank vector (con) and shOrai1 plasmids were introduced into FDB muscle of the living mice using an electroporation method [35]. As shown in **Fig. 2C**, 2 weeks after gene delivery, more than 50% of the muscle fibers in the FDB were RFP-positive. Individual muscle fibers were isolated and RFP-positive fibers were picked using a capillary tube for western blot analysis [36] of Orai1 protein expression. These experiments revealed significant knock down of the endogenous Orai1 by the shOrai1 probes in transfected *wt* and *mdx* fibers (**Fig. 2D, 2E and 2F**), proving the *in vivo* efficacy of the shRNA probes. Furthermore, this shRNA probe had no effect on the expression of STIM1, thus any effects on SOCE could not be a result of off-target knockdown of other major component of the SOCE molecular machinery. The efficiency of our shRNA probes against orai1 had recently been confirmed in a separate study to knock down Orai1 in cultured HL-1 cardiomyocytes [37].

**Figure 3:**
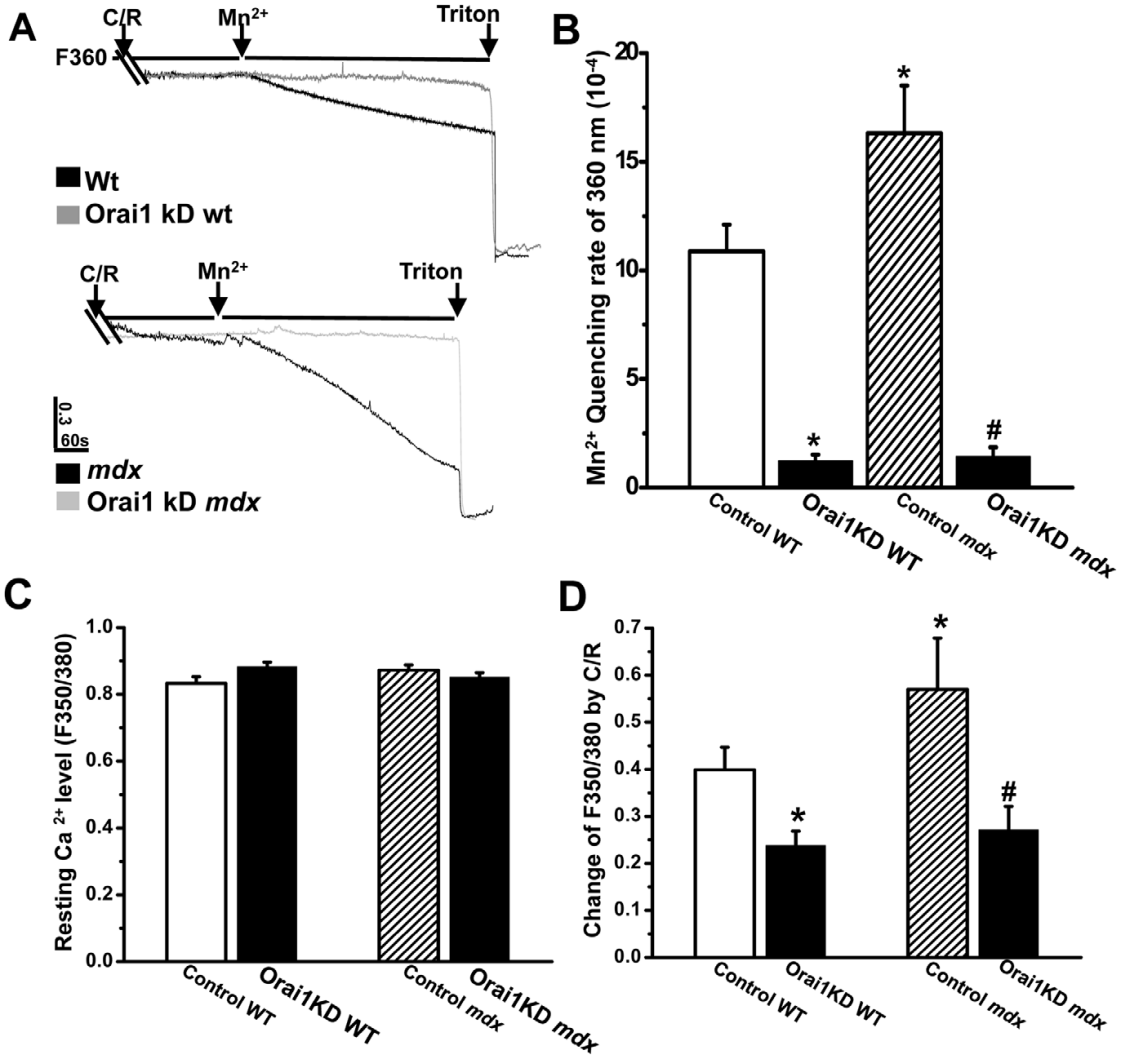
Elevation of Orai1-mediated SOCE activity and SR Ca^2+^ store overload in adult *mdx* muscles. (**A**) Representative trace of Mn^2+^ quenching of Fura-2 fluorescence at 360 nm (F_360_) wavelength. Lines and arrows designate perfusion of muscle fiber by 20 mM caffeine plus 5 uM ryanodine, MnCl_2_ and TritonX-100. Upper panel: black trace is FDB fiber from *wt* mice transfected with empty vector (control WT) and grey trace is with shOrai1 (Orai1KD WT); lower panel: black trace is FDB fiber from *mdx* mice transfected with empty vector (Control *mdx*) and grey trace is with Orai1 (Orai1KD *mdx*). (**B**) Statistical summarization of the data in (A), n = 11–19, ^*^ *P*<0.05 compared to Control WT; ^#^ *P*<0.05 compared to Control *mdx*. (**C**) Statistical results of resting intracellular Ca^2+^ levels in Control WT (open bar), Orai1KD WT (black bar), Control *mdx* (hatched bar) and Orai1KD *mdx* (black bar). (**D**) Statistical results of caffeine-sensitive SR Ca^2+^ store in the four groups. n = 11–19, ^*^ *P*<0.05 compared to Control WT; ^#^ *P*<0.05 compared to Control *mdx*.

The functional consequences of reduced Orai1 expression was assessed by measuring Mn^2+^ quenching of Fura-2 fluorescence to detect SOCE activity in intact adult skeletal muscle fibers [38]. As shown in **Fig. 3A and 3B**, Ca^2+^ entry following SR store depletion by caffeine plus ryanodine was significantly higher in the control *mdx* muscle fibers than the control *wt* FDB fibers. Knock down of Orai1 abolished majority of the SOCE activity in both *wt* and *mdx* muscle fibers. These findings indicate that the Orai1 pore-conducting unit constitutes a major source of Mn^2+^-sensitive SOCE in adult skeletal muscle fiber despite distinct Ca^2+^ entry kinetics between the excitable and non excitable cells [34]. Furthermore, a significant portion of the excessive Ca^2+^ entry apparent in *mdx* fibers may pass through the amplified SOCE pathway since Orai1 knockdown reduced the excessive Ca^2+^ entry in *mdx* fiber to a level comparable to that of the *wt* fiber. However, our results cannot exclude the possibility that Ca^2+^ entry from other sources, including Ca^2+^ leak channels, receptor activated Ca^2+^ entry and additional Ca^2+^ entry pathways, may also contribute to the Ca^2+^ deregulation in dystrophic muscles [13], [39].

Early studies detected a greater level of global Ca^2+^ concentration in muscle biopsies from DMD patients than that seen in healthy volunteers [40], but the level of [Ca^2+^]_i_ detected in *mdx* muscle fibers has varied in different studies [41]. In these preparations we did not resolve a significant difference in the resting Ca^2+^ level between the *wt* and *mdx* fibers using radiometric measurement of Fura-2 fluorescence (**Fig. 3C**), suggesting dystrophic fibers maintain normal control of [Ca^2+^]_i_ by the SR Ca^2+^ uptake and sarcolemmal extrusion systems [42]. This may be a distinct aspect of *mdx* muscle fibers that contributes to the relatively mild symptoms of the *mdx* mice when compared to human DMD patients. Additionally, it is possible our use of Fura-2 fluorescent dye may not detect changes in certain microdomains of the fiber or subtle alteration to overall [Ca^2+^]_i_ levels since the dye itself provides significant buffering of free Ca^2+^ in the cytosol.

Other investigators previously showed that the action potential-induced Ca^2+^ transient is significantly lower in *mdx* muscle fiber while others found that the SR Ca^2+^ store in *mdx* muscle is significantly higher than in controls [43]. In this study, we applied combination of caffeine and ryanodine to clamp the RyR channel in its open state, so the total SR Ca^2+^ store can be assessed. Our result found that the C/R-releasable SR Ca^2+^ store was significantly higher in the *mdx* muscle than in *wt* muscle (**Fig. 3D**), possibly as a result of elevated SOCE. This combination of increased SOCE and an overloaded SR Ca^2+^ store in *mdx* results in an increased overall Ca^2+^ burden in the dystrophic fiber, which may be close to the maximal compensatory capacity of the muscle that will eventually result in death of the muscle fiber. The SR Ca^2+^ overload in *mdx* muscle can be rescued by knock down of Orai1 (**Fig. 3D**), suggesting that upregulated SOCE machinery likely is the causative factor for the Ca^2+^ store overload in *mdx* muscle. Furthermore, knocking down of Orai1 in *wt* control also reduces the SR Ca^2+^ store in transfected muscle fibers (**Fig. 3D**), confirming the role of Orai1-mediated SOCE in replenishing the SR Ca^2+^ store as part of normal muscle physiology [44].

We previously examined the osmotic-shock induced Ca^2+^ spark activity in *mdx* muscles [45], where the Ca^2+^ sparks localized at sarcolemmal periphery in *wt* fibers are found within the fiber center and also appear at higher frequency in *mdx* muscle. These results have been confirmed by studies from other investigators [46], [47]. Our findings here suggest that overloading of the SR Ca^2+^ store in the *mdx* muscle fibers may provide the source for the increased Ca^2+^ spark activity in these fibers. Taken together, our data showed that dystrophic muscle fibers of 8 to10 weeks are facing an increased Ca^2+^ burden throughout different cellular compartments yet the cytosolic Ca^2+^ level is still tightly maintained.

**Figure 4:**
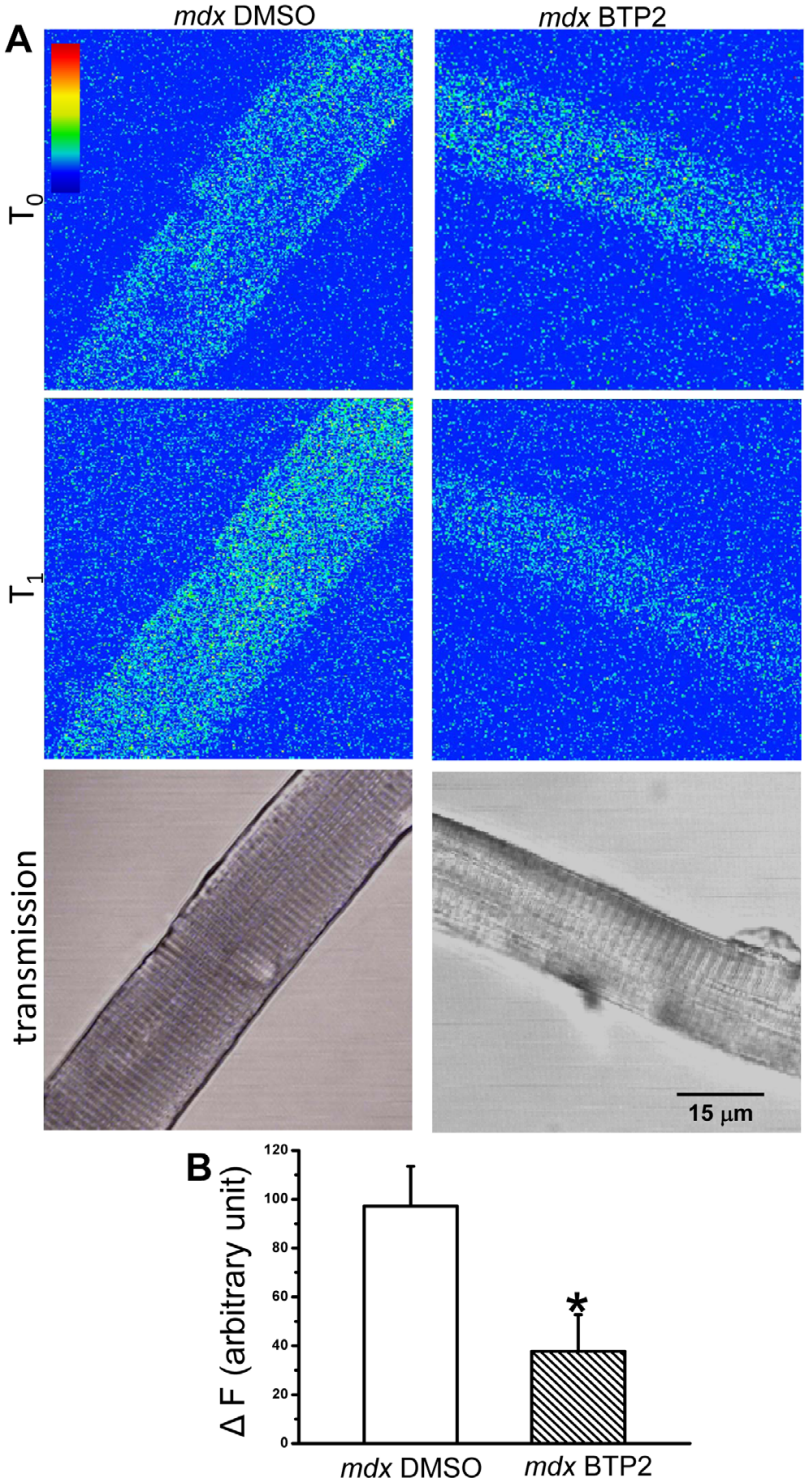
SOCE inhibitor reduces calpain activation in dystrophic fibers. (**A**) Confocal images of FDB fibers from DMSO-injected *mdx* mice and BTP2 injected *mdx* mice at T_0_ (addition of 50 µM fluorogenic, membrane-permeable peptide, Suc-LLVY-AMC) and T_1_ (25 min later to allow for penetration of Suc-LLVY-AMC into FDB fiber and cleavage by cytosolic calpain) at excitation wavelength of 360 nm and emission wavelength of 460 nm. The lower transmission panel is to show that only healthy muscle fibers with clear striation pattern were chosen for the experiment; (**B**) Statistical analysis of changes in the fluorescence signals (ΔF) generated by Suc-LLVY-AMC cleavage in control *mdx* fiber (open bar) and BTP2-treated *mdx* fiber (hatched bar), n = 8 (BTP2) and 14 (DMSO), ^*^ *P*<0.05.

The enzymatic activity of calpain is significantly increased in mouse and human dystrophic muscle, which may be related to the increased proteolysis in dystrophic muscle and progresses of muscular dystrophy [33], [48]–[50]. To probe the contribution of Orai1-mediated Ca^2+^ entry in calpain activation, we adopted a method using a fluorogenic, membrane-permeable peptide, Suc-LLVY-AMC to measure the relative enzymatic activity of calpain. As shown in **Figure 4**, intraperitoneal injection of BTP-2, a specific SOCE inhibitor, for 2 weeks significantly reduced the calpain activity in *mdx* muscle fiber, as compared to the mice injected with vehicle control. This indicates that even without elevation in the steady state cytosolic Ca^2+^ concentration, increase in Ca^2+^ influx through SOCE can activate calpain in dystrophic muscle fibers.

**Figure 5:**
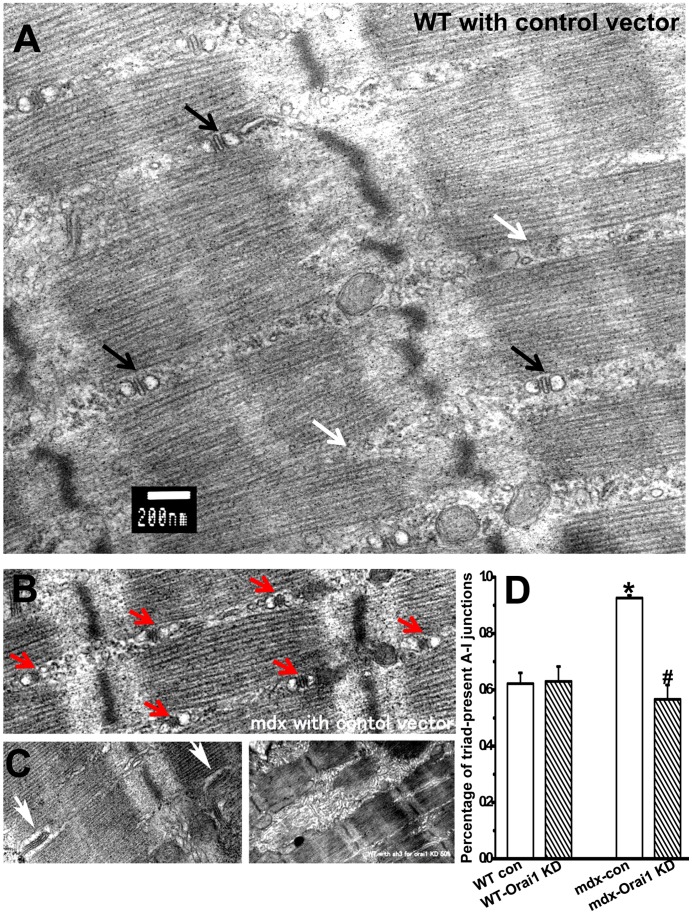
Changes in muscle ultrastructure after Orai1 knockdown. Longitudinal section of FDB muscles under transmission EM of the (**A**) *wt* muscle transfected with control vector. Scale bar is 200 nm. Black arrows designate triads at A-I junctions and white arrows designate triad-defect A-I junctions; (**B**) *mdx* muscle transfected with control vector. Red arrows designate the dense triads at A-I junctions; (**C**) abnormal features observed in muscles with Orai1 knockdown. Left: disoriented triad junctional structure (white arrows); right: thick and rough reticulation as compared to the fine SR network structure in *wt* (A) and *mdx* control (B) muscle; (**D**) Statistical results summarize the percentage of triad-containing A-I junctions in muscle bundles from *wt* control, *wt* Orai1 knockdown, *mdx* control and *mdx* Orai1 knockdown. Data are mean ± S.E.M. *P<0.05 compared to *wt* control and ^#^ *P*<0.05 compared to *mdx* control.

We next examined the ultrastructural alterations of the *wt* and *mdx* muscle with Orai1-knockdown using electron microscopy (EM). As shown in **Fig. 5A**, longitudinal sections of the *wt* muscle reveal ordered sarcomeres and clear triad structure at A-I junctions. The structure of SR is apparently abnormal in *wt* muscle with Orai1 knockdown (**Fig. 5C**), showing thick and rough reticulation and disoriented terminal cisterna structures. This suggests that Orai1 expression can influence SR ultrastructure in skeletal muscle, either through direct interaction with STIM1 in the SR [51], [52] or because that reduced SR Ca^2+^ storage following Orai1 knockdown compromises SR morphology. Our data is the first piece of evidence to show the ultrastructural changes that result from decreased Orai1 expression in muscle. The overall ultrastructure of the observed *mdx* muscle fibers appears generally normal (**Fig. 5B**), consistent with previous studies [53], [54]. However, upon careful quantification, we found the number of triads at A-I junctions was significantly increased in the *mdx* muscles (**Fig. 5D**). It is possible that the increased triad junctions may reflect a compensatory mechanism to manage the elevated SR Ca^2+^ load in *mdx* muscle or an indication of muscle regeneration.

**Figure 6:**
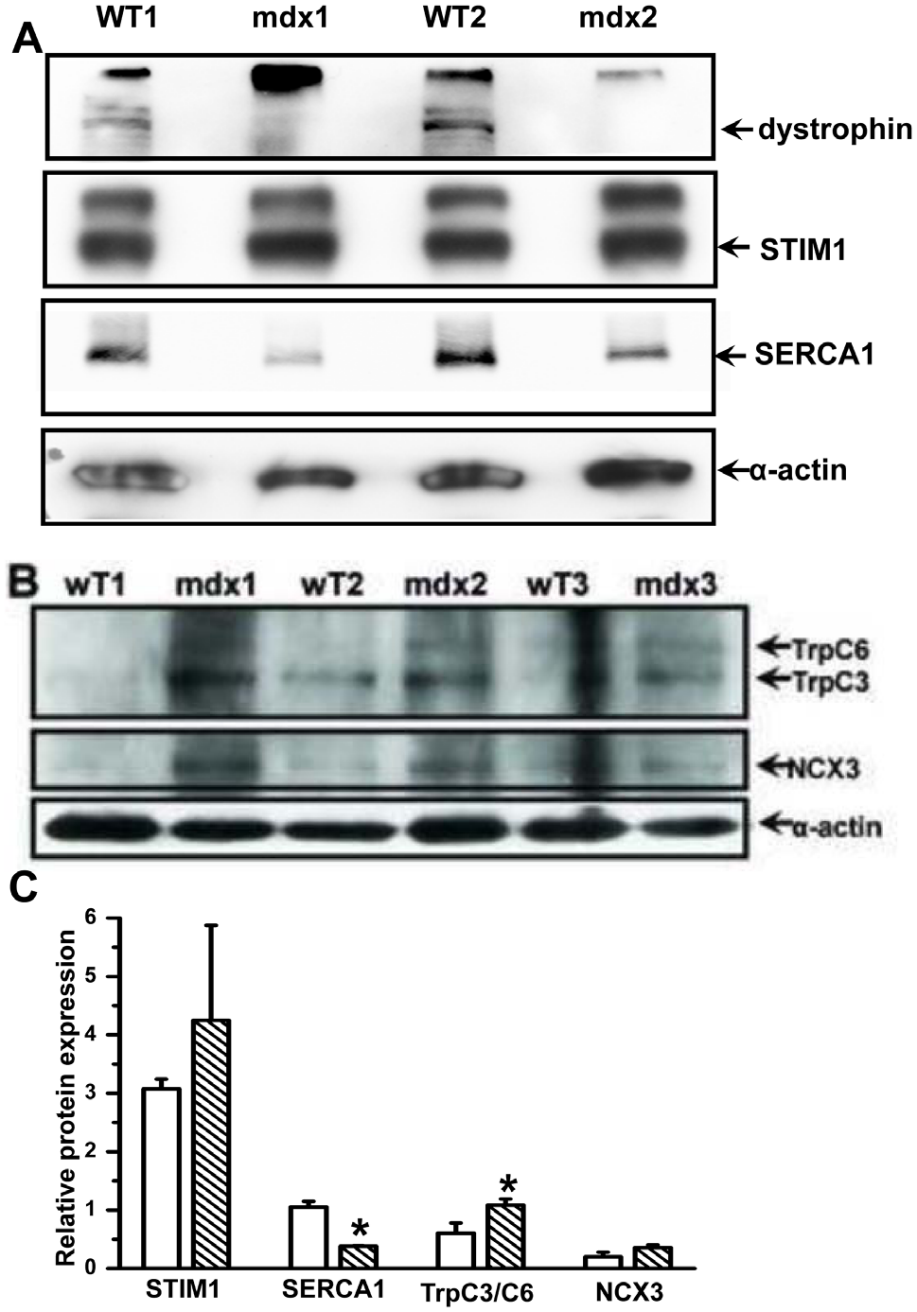
Compensatory change in protein expression of other Ca^2+^ shuttling pathways in *mdx* muscles. (**A**) Absence of the 427 kDa dystrophin in *mdx* muscles was confirmed and expression levels of STIM1 and SERCA1 in FDB muscles from the *wt* C57BL/10ScSnJ and dystrophic C57BL/10ScSn-Dmd*^mdx^*/J mice were tested by western blot. Sarcomeric a-actin was used as a loading control. (**B**) In a separate study, the levels of TRPC3/6 and NCX3 were tested in *wt* and *mdx* muscles. (**C**) Densitometry of detected protein expression relative to α-actin, n = 2 for STIM1 and SERCA1 and n = 3 for TrpC3/C6 and NCX, *P<0.10.

To further elucidate additional adaptive or compensative responses of the dystrophic muscle to the increased Ca^2+^ burden following elevated SOCE we assessed the protein levels of other essential Ca^2+^ signaling proteins in skeletal muscle by performing western blots for STIM1 and SR/ER Ca^2+^ ATPase (SERCA) 1. As shown in **Fig. 6A**, we frequently detected two bands by the STIM1 antibody, one at the predicted size for STIM1 (80 kDa) and another at 100 kDa, possibly representing a post-translational modification of the STIM1 protein. The *wt* and *mdx* muscles displayed similar levels of STIM1 expression, indicating that skeletal muscle fibers contain a surplus of STIM1 beyond that which is necessary to activate Orai1. This idea is supported by a recent study showing multiple functions for STIM1 in skeletal muscles, including activation of SOCE and inhibition L-type Ca^2+^ channel function simultaneously [55]. In addition, the protein expression level of SERCA1 was significantly reduced in *mdx* muscle as compared to that of the control muscle (SERCA1/α-actin densitometry value of 0.65±0.08 vs. 0.16±0.04, n = 6∼7 and *P*<0.001). It is possible that the altered SERCA expression may be an adaptive change to the increased SR Ca^2+^ load in the *mdx* muscle fiber. The expression level of transient receptor potential channel (TRP) C3/6, the molecular components for another potential Ca^2+^ entry pathway in skeletal muscle [39], [56], was also tested here (**Fig. 6B**). Our antibody detected a TRPC6 band at ∼111 kDa and a TRPC3 band at ∼97 kDa. The endogenous expression level of either isoform of TRPC appears to be relatively low in skeletal muscles, consistent with previous reports [39], [56]. However, there appears to be an increase in TRPC3/6 expression in the *mdx* muscle preparation (**Fig. 6C**), suggesting possible additional source for the increase Ca^2+^ entry in mdx skeletal muscles. In addition, we probed the expression of the skeletal muscle isoform sodium-calcium exchanger, i.e., NCX3 and our data showed slight upregulation in NCX3 expression (**Fig. 6B and 6C**). However, the difference in NCX3 expression was not statistically significant between the *wt* and the *mdx* skeletal muscle.

**Figure 7:**
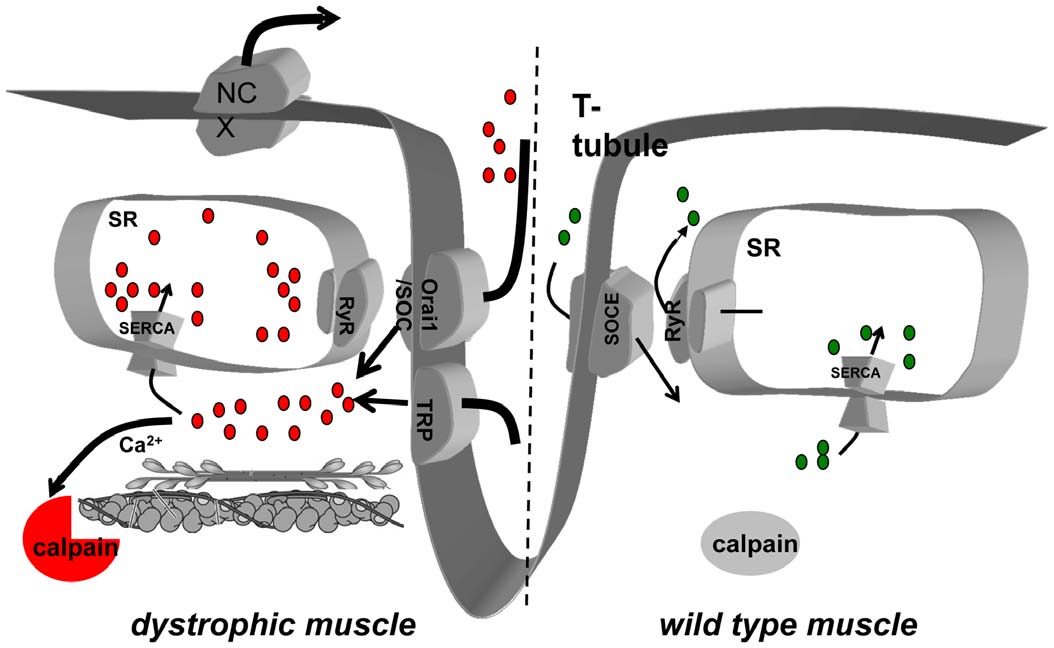
In *mdx* dystrophin null muscle fibers elevated Ca^2+^ entry is at least partially mediated by Orai1 upregulation, leading to an increased SR Ca^2+^ store. The excessive Ca^2+^ entry contributes to activate calpain in the cytosol and possibly leads to increased proteolytic activity. An adaptive downregulation in SERCA1 expression then occurs to accommodate overloaded SR Ca^2+^ store and upregulation in NCX expression to maintain the low level of resting cytosolic Ca^2+^ level.

Overall, our results indicate that in dystrophic muscle fibers, upregulation in Orai1 expression is one pathway leading to excessive Ca^2+^ entry that causes overloading of the SR Ca^2+^ store and an accompanying reduction in SERCA1 expression. The elevated Ca^2+^ entry in dystrophic muscle is linked to an increased in cytosolic calpain activity, which may contribute to the progression of muscular dystrophy (**Fig. 7**).

## Discussion

A significant body of evidence indicates that dysregulation of Ca^2+^ influx plays a key role in the pathogenesis and progression of DMD [57] and many candidates have been proposed to mediate the elevated Ca^2+^ entry in dystrophic fibers, such as stretch-activated channels (SACNSC) [58] and several members of the TRP channel family [39]. Nearly 10 different TRP channels isoforms have been detected in skeletal muscle by RT-PCR, western blot or immunohistochemistry [59], and here we confirm that TRPC3 and TRPC6 were expressed in skeletal muscle (**Fig. 6B**). TRPC function may contribute to the progression of muscular dystrophy as previous studies showed that by expressing a dominant negative TRPC6 could reduce the pathology in *mdx* and sarcoglycan null mice [39]. Here we detected that the expression level of Orai1 protein expression in dystrophic muscle was increased by ∼2-fold while the ER Ca^2+^ sensor STIM1 remain unchanged (**Fig. 6A** and **Fig. 1**). We also found that the expression level of TRPC3/6 was increased in dystrophic muscle (**Fig. 6B and 6C**). Given previous studies showing that several isoforms of TRP channel form hetero-multimers with Orai1, including TRPC1, 3 and 4, to modulate Orai1 function in SOCE [60], we speculate the excessive TRPC expression may contribute to the activation of Orai1-depedent SOCE independent of the SR store status. This could contribute to the elevated Ca^2+^ entry observed in *mdx* muscle even though we determined the Ca^2+^ store was overloaded in these fibers (**Fig. 3D**). Under these conditions, the Ca^2+^-sensing mechanism of the SOCE pathway may be disengaged and the elevated TRPC levels could directly activate Orai1 function. This idea is generally supported by previous immunohistochemical data by Krüger et al. [61] that shows TRPC6 localized at the sarcolemma and TRPC3 staining in intracellular patches preferentially in *mdx* muscle [**61**], suggesting that different TRPC isoforms may have various functions in the regulation of Orai1 function.

We have shown here that the Orai1 expression level was up-regulated in dystrophic muscles, consistent with a report by Edwards et al. [62]. The functional analysis performed by this group showed an ultra-fast activation and deactivation of SOCE induced by change in Mg^2+^ concentration in skinned muscle preparations, a response with kinetic properties in excess of that previously observed in intact muscle preparations [63], [64]. In this study, we systematically studied the contribution of Orai1-mediated Ca^2+^ entry in the progression of muscular dystrophy by applying a shRNA targeting Orai1 and systematic inhibition of SOCE using BTP2. Our results suggest that Orai1 upregulation causes gain of function in SOCE in *mdx* muscle fiber, leading to overload of the SR Ca^2+^ store. Treatment by BTP2 also significantly reduced the calpain enzymatic activity in dystrophic muscle fibers, which was linked to the increased proteolytic events that eventually result in progression of the dystrophic phenotype [41], [65]–[67]. Thus, the current data suggest that dystrophic muscle fibers of 8 to 10 weeks are facing an increased Ca^2+^ burden throughout different cellular compartments although the cytosolic Ca^2+^ level is still tightly maintained. Even without elevation in resting cytosolic Ca^2+^ concentration, increase in Ca^2+^ influx through SOCE can still activate calpain that would result in degradation of contractile proteins in the dystrophic muscle fiber. We have also recently found Orai1 can contribute to Ca^2+^ entry in cultured HL-1 cardiomyocytes [37], suggesting that Orai1 could potentially contribute to physiology and pathophysiology in other striated muscle tissue.

The typical histological alterations of muscular dystrophy includes reduced cross-section area, increased central nucleus and isolated fibrosis, indicating the constant process of muscle damage and regeneration [68]. In *mdx* mice, the pathology of affected muscles varies with the age of the animal. Such mice display dystrophic symptoms after the first few weeks of life and then recover through fiber regeneration over the next few weeks. Therefore, we examined *mdx* mice at 8 to 10 weeks of age for our study to minimize the heterogeneity of the structural alterations [68]. Our EM data showed that even during this stage, the overall ultrastructure of non-regenerating dystrophic muscle fiber is largely intact. However, upon careful analysis of the SR presented at A-I junctions, we found that dystrophic muscle had significantly increased quantity of SR (**Fig. 5D**). It is possible that the increased triad junctions may reflect compensation to manage the elevated SR Ca^2+^ load in *mdx* muscle or be one of the subtle indications for muscle regeneration. The shRNA probe used in our study could effectively knockdown the expression of Orail (**Fig. 2**), which led to disoriented triad structure and rough SR network of the skeletal muscle (**Fig. 5C**), indicating that Orai1 expression is required to maintain the structural integrity of the muscle triad junctional complex. Our data is the first piece of evidence to show the ultrastructural changes that result from decreased Orai1 expression in muscle.

In the current study we also explored the expression level of other Ca^2+^ handling proteins in *mdx* muscle. A significant decrease in protein expression of SERCA1 was detected in skeletal muscles from the *mdx* mice. However, since no increase in resting cytosolic Ca^2+^ levels was seen in the dystrophic fibers the remaining SERCA must be sufficient to sequester the excessive cytosolic Ca^2+^ produced by SOCE into the SR and overload the Ca^2+^ store (**Fig. 3C**). Our findings here indicate that recent studies showing partial rescue of the phenotype of *mdx* mice by overexpressing SERCA1 specifically in the skeletal muscle may involve replacing lost SERCA1 expression [69]. It is also possible that the decrease in SERCA1 expression may be an adaptive change to the overloaded SR Ca^2+^ store in *mdx* fibers. A recent report by Robin et al. revealing increased SERCA1 activity in *mdx* muscle fibers is consistent with the idea of a compensatory changes in SERCA function in dystrophic muscle [70].

In summary, our results suggest that elevated Ca^2+^ entry follows Orai1 upregulation in *mdx* dystrophic muscle fibers, leading to an overfilled SR Ca^2+^ store (**Fig. 7**). The excessive Ca^2+^ entry activates calpain in the cytosol and possibly leads to increased proteolytic activity. An adaptive downregulation in SERCA1 expression then occurs to accommodate overloaded SR Ca^2+^ store and slight upregulation in NCX expression to maintain normal cytosolic Ca^2+^ levels. Although a previous studies suggests a link between extracellular Ca^2+^ entry and the cytosolic [Ca^2+^] level [71], we cannot detect a change in [Ca^2+^]_i_ under these experimental conditions. Our study indicates that excessive Ca^2+^ entry through Orai1 may underlie Ca^2+^-mediated muscle damage during DMD progression and could represent a potential therapeutic target for the treatment of DMD. Additional studies to investigate the potential mechanisms for increased Orai1 expression following absence of dystrophin are of high interest to elucidate the underlying mechanism for the progression of DMD.

## Materials and Methods

### Design of shRNA Probes and Construction of Vectors

Three shRNA probes specifically targeting mOrai1 CDs (NM_175423) were designed. The oligonucleotide sequences for sh2 and sh3 were as follows: sh2: sense: 5′-GAT CGT CCT GGC GCA AGC TCT ACT TAA TTC AAG AGA TTA AGT AGA GCT TGC GCC AGG ACT TTT TT-3′; antisense 5′-AAT TAA AAA AG T CCT GGC GCA AGC TCT ACT TAA TCT CTT GAA TTA AGT AGA GCT TGC GCC AGG AC-3′ and sh3: sense: 5′-GAT CGT GCA CCT GTT TGC CCT CAT GAT TTC AAG AGA ATC ATG AGG GCA AAC AGG TGC ACT TTT TT-3′, and antisense 5′-AAT TAA AAA AGT GCA CCT GTT TGC CCT CAT GAT TCT CTT GAA ATC ATG AGG GCA AAC AGG TGC AC-3′. These shOrai1 probes were cloned into a custom-made pU6r-RFP vector with a multi-red fluorescence protein (RFP) expression cassette. Scramble shRNA in pU6r-RFP vector was used as control for all experiments. Full-length mOrai1 cDNA in pcDNA3.1/Myc-His(−) vector (Invitrogen) was co-transfected with sh2 and sh3 into HeLa cells to test the efficiency of Orai1 knockdown.

### Cell Culture and Transfection

HeLa cells were cultured in Dulbecco’s modified Eagle’s medium supplemented with 10% fetal bovine serum, 100 units/ml penicillin and 100 µg/ml streptomycin at 37°C in an incubator suffused with 5% CO_2_. Three shRNA probes targeting mOrai1 in pU6r-RFP plasmids were co-transfected with full length mOrai1 pcDNA3.1/Myc-His(−) at 9∶1 molar ratio into HeLa cells using GeneJammer transfection reagent (Stratagene, Cedar Creek, TX) (2 µg DNA: 4 µl reagent) according to the manufacturer’s instructions. Experiments were repeated twice. HeLa cells were used for these experiments as these easily transfected cells allows for resolution of the degree of knockdown without complication from the lower levels of transfection seen in some immortalized muscle cell cultures.

### Animals

All animal work was conducted according to relevant national and international guidelines. The full details of this study were reviewed and approved by the Robert Wood Johnson IACUC. 6 week old Male C57BL/10ScSnJ (*wt*) and C57BL/10ScSn-Dmd*^mdx^*/J dystrophic mice (*mdx*) were purchased from Jackson Laboratory (Bar Harbor, ME). Animals were bred and housed in the Robert Wood Johnson Medical School vivarium facility until they were 8∼10 weeks old before use in our experiments.

### Electroporation of Flexor Digitorum Brevis (FDB) Muscle Fiber

The control plasmid and shOrai1 probes were introduced into the FDB muscles of 8 weeks old *wt* or *mdx* mice using an electroporation procedure as previously described [35]. Briefly, the animal was anaesthetized with ketamine and xylazine before subcutaneous hyaluronidase injection into each rear foot pad. After 1 hour, control or shOrai1 plasmid DNA in sterilized 0.9% saline was injected into muscle and two acupuncture needles (Austin Medical Equipment, Westchester, IL) connected with the anode and cathode of an ECM 830 Electroporator (BTX, Holliston, MA) were inserted subcutaneously into the FDB muscle. A series of 20 ms square wave pulses of 120 V/cm were generated to promote DNA entry into FDB muscle fibers.

### Real-time PCR

Total mRNA was extracted from gastrocnemius muscles of the C57BL/10ScSnJ and dystrophic C57BL/10ScSn-Dmd*^mdx^*/J mice using a RNAasy kit and on-column genomic DNA clean kit (Qiagen, Valencia, CA). Isolated mRNA was quantified using UV spectrometer and verified by formaldehyde denaturing agarose gel electrophoresis. Real-time PCR for Orai1, STIM1 and Glyceraldehyde 3-phosphate dehydrogenase (GAPDH) was performed using our previously described primers and protocols [32]. Triplicate wells were used for each sample and the relative number of mRNA copies to GAPDH was calculated using the formula: 1/2^Λ (ct value of target gene – ct value of GAPDH)^.

### Western Blot Assay

Effective knock down of Orai1 by shRNA *in vivo* was confirmed by western blot using extracts from individual FDB fibers [36]. Briefly, electroporated FDB bundles were enzymatically dissociated and individual transfected single muscle fiber was collected by a 0.8–1.1 mm × 100 mm capillary tube (PYREX, Lowell, MA) under Zeiss Axiovert 2000 fluorescence microscope (Zeiss, Thornwood, NY) and pooled for Western blot assay. Cultured cells, intact muscle bundles or pooled single muscle fibers from *wt* and *mdx* mice were lysed in a highly reducing RIPA buffer (in mM, 150 NaCl, 20 NaPO_4_, 50 NaF, 2 EDTA, 30 Na pyrophosphate, 1 phenylmethanesulphonylfluoride, 0.2 Na vanadate, 14 β-mercaptoethanol, 100 dithiothreitol, 0.1% SDS, 1% deoxycholic acid, 1% triton X-100, 100 KIU/ml aprotinin and 1% p8340 protease inhibitor cocktail (Sigma, St. Louis, MO), pH 7.2) and separated by electrophoresis on a 12% SDS gel. After transfer, PVDF membranes were probed with anti-Orai1 polyclonal (1∶1,000 dilution, Millipore, Billerica, MA), anti-myc (1∶1,000), anti-STIM1 (1∶500) monoclonal (BD Bioscience, Franklin Lakes, NJ), anti-dystrophin monoclonal (1∶500, DSHB, Iowa City, Iowa), anti-SERCA1 monoclonal (1∶8,000, ABR, Golden, CO), anti-NCX3 polyclonal (1∶1,000, Santa Cruz, CA), or TRPC3/6 (1∶1,000, generous gift from Dr. Michael Zhu, University of Texas Health Science Center at Houston). Sarcometirc anti-α-actin (1∶8,000) and β-actin antibodies (Sigma, St. Louis, MO) were used as loading controls. Densitometry of target proteins was performed and normalized to loading controls using Genetools software (Syngene, Frederick, MD).

### Ca^2+^ Measurement and SOCE Activity by Mn^2+^ Entry Assay

The procedure was described in detail previously [38]. Briefly, transfected FDB muscle fibers were enzymatically disassociated and loaded with 10 µM Fura-2 AM (Molecular Probes, Eugene, OR). To prevent motion artifact associated with increase in [Ca^2+^]_i_, 30 µM N-benzyl-p-toluene sulphonamide (Sigma, St. Louis, MO) was applied on muscle fiber for 15 min and left on during the experiment. FDB fibers were examined on a PTI spectrofluorometer system (Photon Technology International, Monmouth Junction, NJ) and selected by presence of RFP signal under wavelength of 550 nm. Fura-2 fluorescence ratio of excitation wavelengths at 350 nm (F_350_) and 380 nm (F_380_) and emission at 510 nm was recorded for measuring SR Ca^2+^ store induced by 20 mM caffeine plus 5 µM ryanodine (C/R). Mn^2+^ quenching of Fura-2 fluorescence was performed at a wavelength of 360 nm following depletion of SR store by C/R dissolved in 0 mM Ca^2+^. 1% Triton X-100 was added at the end of the experiment for data normalization. All experiments were conducted at room temperature.

### Determination of Calpain Activity in situ

To inhibit SOCE activity, *mdx* mice were first injected with BTP2 (EMD Biosciences, Rockland, Massachusetts), at a dose of 4 mg/kg body weight, daily Intraperitoneal injection for 3 weeks. Then the mice were sacrificed and enzymatic activity of calpain in single intact FDB fibers was measured using a fluorogenic, membrane-permeable calpain substrate, Succinyl-Leu-Leu-Val-Tyr-7-amino-4 methylcoumarin (Suc-LLVY-AMC) (Enzo life sciences, Farmingdale, NY) based on previous publications [72], [73]. Specifically, enzymatically isolated FDB fibers were plated on glass bottom ΔT dish and treated with 10 µM Lactacystin (Boston Biochem, Cambridge, MA) to inhibit proteasome activity. Then 50 µM Suc-LLVY-AMC was added to the medium and fluorescence signal was monitored immediatedly on Zeiss LSM 510 confocal microscope (Carl Zeiss, Thornwood, NY) using 360 nm excitation and 460 nm emission wavelengths. Measurement of fluorescence signal was continued for 25 min and the relative calpain activity (ΔF) was calculated as peak fluorescence activity (F1) substrates the basal fluorescence level (F0).

### Transmission Electron Microscopy

FDB muscle preparations were pre-fixed with 2.5% glutaraldehyde and 3% formaldehyde in 0.1 M cacodylate buffer (pH 7.4), and post-fixed with 1% OsO_4_ in the same buffer. After washing with cacodylate buffer solution, they were dehydrated with ethanol plus acetone and embedded in epoxy resin. Ultrathin sections were cut, double stained with uranyl acetate and lead citrate, and examined under JEM-1010 electron microscope (Jeol Co., Ltd, Tokyo, Japan).

### Statistics

Values are mean ± S.E.M. Significance was determined by Student’s *t* test. A value of *P*<0.05 was considered to be statistically significant.

## Supporting Information

Figure S1 **Increased Orai1 expression in various muscles from the** ***mdx*** **mice.** Upper panel: Orai1 expression is increased in *extensor digitorum longus* (EDL) of the *mdx* mice, a mostly fast glycolytic skeletal muscle. Blot of α-actin was to show the equal loading of these individual muscles; lower panel: Orai1 expression is increased in gastrocnemius (GN) muscle from the mdx mice, a mixed type of skeletal muscle, as compared to the wild type control.
(TIF)
