# Supplementary figures and images for: Orai1 Mediates Exacerbated Ca2+ Entry in Dystrophic Skeletal Muscle
Source: PLoS One. 2012 Nov 19;7(11):e49862. doi: 10.1371/journal.pone.0049862 (PMC3501460; doi:10.1371/journal.pone.0049862)

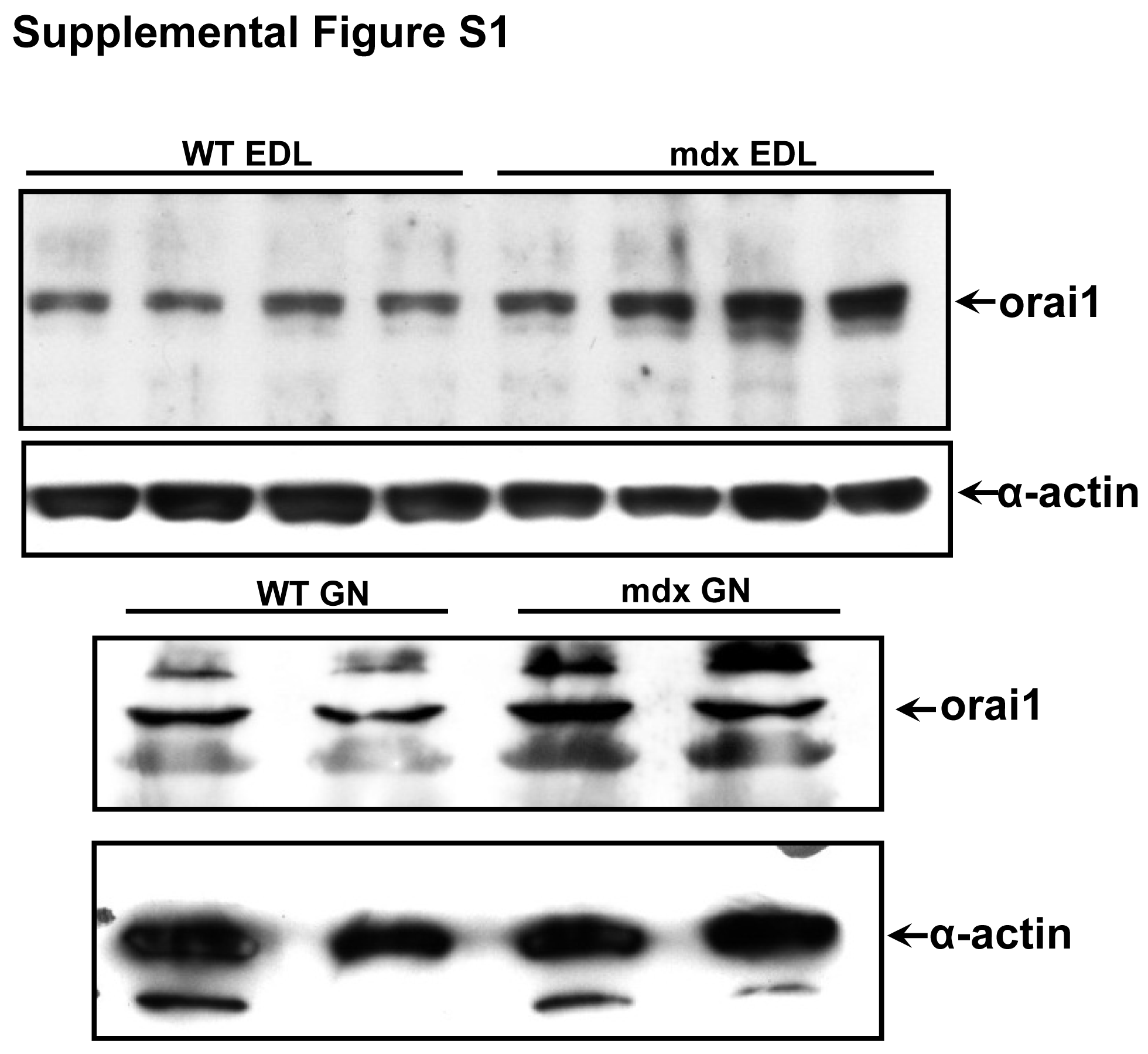

Supplement: Figure S1 — Increased Orai1 expression in various muscles from the mdx mice. Upper panel: Orai1 expression is increased in extensor digitorum longus (EDL) of the mdx mice, a mostly fast glycolytic skeletal muscle. Blot of α-actin was to show the equal loading of these individual muscles; lower panel: Orai1 expression is increased in gastrocnemius (GN) muscle from the mdx mice, a mixed type of skeletal muscle, as compared to the wild type control. (TIF) [file pone.0049862.s001.tif]
